# Supplementary material for: Importin-9 wraps around the H2A-H2B core to act as nuclear importer and histone chaperone
Source: eLife. 2019 Mar 11;8:e43630. doi: 10.7554/eLife.43630 (PMC6453568; doi:10.7554/eLife.43630)
Supplement: Figure 1—source data 1. [file elife-43630-fig1-data1.docx]

**Figure 1- source data 1.** Data collection and refinement statistics, Imp9•H2A-H2B structure.

| **Data collection** | | | |
| --- | --- | --- | --- |
| Crystal | SeMet peak^a^ | | Native |
| Space group | P2_1_2_1_2 | | P2_1_2_1_2 |
| Cell constants (Å) | a = 127.83, b = 223.61, c = 132.35 | | a = 127.42, b = 223.29, c = 131.83 |
| Wavelength (Å) | 0.97940 | | 0.97938 |
| Resolution range (Å) | 45.40 – 2.65 (2.70 – 2.65) | | 45.40 – 2.70 (2.75 – 2.70) |
| Unique reflections | 105,074 (4,974) | | 103,239 (5,166) |
| Multiplicity | 7.3 (5.6) | | 10.1 (8.2) |
| Data completeness (%) | 99.5 (95.1) | | 97.7 (98.9) |
| *R*_merge_ (%)^b^ | 9.1 (100.0) | | 9.4 (208.6) |
| *R*_pim_ (%)^c^ | 4.7 (100.0) | | 2.9 (72.0) |
| CC_1/2_ | 0.52 | | 0.58 |
| I/σ(I) | 14.6 (0.6) | | 20.3 (1.0) |
| Wilson B-value (Å^2^) | 39.8 | | 38.6 |
| **Phase determination** | | | |
| Anomalous scatterers | selenium, 54 out of 60 possible sites | | |
| Figure of merit (45.4 – 2.65 Å) | 0.70 (after density modification) | | |
| **Refinement statistics** | | | |
| Crystal | | Native | |
| Resolution range (Å) | | 45.4 – 2.70 (2.77 – 2.70) | |
| No. of reflections *R*_work_/R_free_ | | 93,884/1,978 (2,186/55 | |
| Data completeness (%) | | 90.5 (31.0) | |
| Atoms (non-H protein, chain A/chain B/ chain C/ chain D/chain E/chain F/water) | | 7,539/673/727/7,444/666/770/356 | |
| *R*_work_ (%) | | 20.9 (30.0) | |
| *R*_free_ (%) | | 24.0 (37.9) | |
| R.m.s.d. bond length (Å) | | 0.002 | |
| R.m.s.d. bond angle (°) | | 0.51 | |
| Mean B-value (Å^2^) (non-H protein, chain A/chain B/chain C/chain D/chain E/chain F/water) | | 50.9/70.3/77.8/46.8/58.0/72.1/35.1 | |
| Ramachandran plot (%) (favored/additional/disallowed)^d^ | | 97.1/2.8/0.1 | |
| Clashscore/Molprobity score^d^ | | 2.43/1.19 | |
| Maximum likelihood coordinate error | | 0.29 | |
| Missing residues | | A: 1-14, 935-996, 1041. B: 1-16, 103-130. C: 1-28, 32-33, 86-87, 125-126. D: 1-14, 234-235, 314-326, 936-996. E: 1-16, 102-130. F: 1-27, 125-126. | |
| PDB Code | | 6N1Z | |

Data for the outermost shell are given in parentheses.

^a^Bijvoet-pairs were kept separate for data processing.

^b^*R*_merge_ = 100 Σ_h_Σ_i_|*I_h,i_*— 〈*I_h_*〉*|/*Σ*_h_*Σ_i_ 〈*I_h,i_*〉, where the outer sum (h) is over the unique reflections and the inner sum (i) is over the set of independent observations of each unique reflection.

^c^ *R*_pim_ = 100 Σ_h_Σ_i_ [1/(n_h_ - 1)]^1/2^|*I_h,i_*— 〈*I_h_*〉*|/*Σ*_h_*Σ_i_ 〈*I_h,i_*〉, where n_h_ is the number of observations of reflections **h**.

^d^As defined by the validation suite MolProbity (Chen, V.B., Arendall, W.B.A., Headd, J.J., Keedy, D.A., Immormino, R.M., Kapral, G.J., Murray, L.W., Richardson, J.S., Richardson, D.C. (2010) *MolProbity*: all-atom structure validation for macromolecular crystallography. *Acta Cryst*. **D66**, 12-21.).
