# Supplementary material for: Importin-9 wraps around the H2A-H2B core to act as nuclear importer and histone chaperone
Source: eLife. 2019 Mar 11;8:e43630. doi: 10.7554/eLife.43630 (PMC6453568; doi:10.7554/eLife.43630)
Supplement: Figure 4—source data 1. [file elife-43630-fig4-data1.docx]

**Figure 4 – source data 1. Summary of the SAXS experiments and analysis**.

| **Data collection** | |
| --- | --- |
| Beamline | SSRL BL4-2, SLAC National Accelerator Laboratory |
| Defining Slits size (mm) | 0.3 (H) × 0.3 (V) |
| Beam energy (keV) | 11 |
| Sample-Detector Distance (m) | 1.7 |
| Detector | Rayonix MX225-HE |
| Pixel binning | 8 × 8 |
| Pixel size (μm) | 292 |
| Exposure time (sec) | 1 |
| Images | 20 images/concentration for duration of run |
| Type of sample cell | Quartz capillary (Diameter = 1.5mm) |
| Temperature (K) | 288 |
| Calibrant | AgBe |
| Final *q* range (Å^-1^) | 0.007 - 0.5 |
| **Data analysis** | |
| Programs | SASTOOL^*^, PRIMUS^*^ |
| Buffer | 20 mM HEPES, 110 mM KAc_2_, 2 mM MgAc_2_, 2 mM DTT, 10% (v/v) glycerol at pH 7.3 |
| Concentration range (mg/ml) | 0.5 - 5.0^**^ |
| Guiner *R_g_* (Å) | 37.8 ± 0.48 (Imp9)  37.3 ± 0.78 (Imp9•H2A-H2B)  36.4 ± 0.59 (Imp9•RanGTP)  37.7 ± 0.44 (RanGTP•Imp9•H2A-H2B) |
| Guiner *qR_g_* limits | 1.30, 1.16, 1.25, 1.30 (same order as above) |
| *D_max_* (Å) | 120.1, 137.6, 126.2, 128.1 (same order as above) |
| Molecular Weight (kDa), SAXS MOW | 124.5, 145.1, 142.7, 161.1 (same order as above) |
| Molecular Weight (kDa), sequence | 115.8, 143.5, 135.5, 163.2 (same order as above) |
| **Modeling** | |
| Programs | DAMMIF, DAMMIN, DAMAVER |
| Data plotting | ATSAS, SAXS MOW, and UCSF Chimera |

^*^SasTool (SasTool, 2013), PRIMUS (Konarev et al., 2003)

^**^Merged SAXS profiles were shown in Figure 4 – figure supplement 3

ATSAS package.
