# Supplementary material for: Importin-9 wraps around the H2A-H2B core to act as nuclear importer and histone chaperone
Source: eLife. 2019 Mar 11;8:e43630. doi: 10.7554/eLife.43630 (PMC6453568; doi:10.7554/eLife.43630)
Supplement: Figure 4—source data 2. [file elife-43630-fig4-data2.docx]

**Figure 4 – source data 2. Summary of the SAXS parameters**.

| **Sample** | **Concentration** | | **SAXS parameters** | | | ***M.W.* from sequence** |
| --- | --- | --- | --- | --- | --- | --- |
|  | mg/ml | μM | *M.W.* (kDa)* | *D_max_* (Å) | *R_g_* (Å)** |  |
| **Imp9** | 0.5 | 4.3 | 120.3 | 116.9 | 37.3 | 115.8 kDa |
|  | 1.0 | 8.6 | 124.7 | 125.5 | 37.7 |  |
|  | 1.5 | 13.0 | 123.8 | 130.1 | 37.7 |  |
|  | 2.0 | 25.9 | 134.0 | 146.1 | 38.1 |  |
|  | 5.0 | 43.2 | 125.5 | 127.3 | 37.0 |  |
|  | **Merged** | | **124.5** | **120.1** | **37.8** |  |
| **Imp9•**  **H2A-H2B** | 0.5 | 3.5 | 151.8 | 125.3 | 37.7 | 143.5 kDa |
|  | 1.0 | 7.0 | 144.3 | 135.4 | 38.4 |  |
|  | 1.5 | 10.5 | 162.3 | 142.7 | 39.3 |  |
|  | 2.0 | 13.9 | 156.1 | 141.1 | 39.6 |  |
|  | 5.0 | 34.8 | 179.0 | 222.6 | 44.3 |  |
|  | **Merged** | | **145.1** | **137.6** | **37.3** |  |
| **Imp9•**  **RanGTP** | 0.5 | 3.7 | 145.6 | 134.7 | 37.1 | 135.5 kDa |
|  | 1.0 | 7.4 | 130.0 | 118.7 | 36.4 |  |
|  | 1.5 | 11.1 | 140.2 | 130.0 | 37.7 |  |
|  | 2.0 | 14.8 | 151.7 | 120.9 | 36.6 |  |
|  | 5.0 | 36.9 | 143.5 | 122.5 | 36.9 |  |
|  | **Merged** | | **142.7** | **126.2** | **36.4** |  |
| **RanGTP• Imp9•**  **H2A-H2B** | 0.5 | 3.1 | 169.5 | 135.0 | 38.4 | 163.2 kDa |
|  | 1.0 | 6.1 | 158.2 | 130.5 | 37.9 |  |
|  | 1.5 | 9.2 | 158.4 | 140.5 | 38.0 |  |
|  | 2.0 | 12.3 | 162.1 | 130.2 | 37.8 |  |
|  | 5.0 | 30.6 | 168.6 | 183.0 | 40.8 |  |
|  | **Merged** | | **161.1** | **128.1** | **37.7** |  |

* Molecular Weights (*M.W.*) were estimated using SAXS MOW with a threshold of *q_max_* = 0.25 ~ 0.3 (1/Å), depending on the data.

** Radius of gyrations (*R_g_*) were calculated in real space using DATGNOM in the ATSAS package.
